# Identification and functional analysis of NOL7 nuclear and nucleolar localization signals

**DOI:** 10.1186/1471-2121-11-74

**Published:** 2010-09-27

**Authors:** Guolin Zhou, Colleen L Doçi, Mark W Lingen

**Affiliations:** 1 Departments of Pathology, Medicine and Radiation and Cellular Oncology, The University of Chicago, Chicago, IL, USA

## Abstract

**Background:**

NOL7 is a candidate tumor suppressor that localizes to a chromosomal region 6p23. This locus is frequently lost in a number of malignancies, and consistent loss of NOL7 through loss of heterozygosity and decreased mRNA and protein expression has been observed in tumors and cell lines. Reintroduction of NOL7 into cells resulted in significant suppression of *in vivo *tumor growth and modulation of the angiogenic phenotype. Further, NOL7 was observed to localize to the nucleus and nucleolus of cells. However, the mechanisms regulating its subcellular localization have not been elucidated.

**Results:**

An *in vitro *import assay demonstrated that NOL7 requires cytosolic machinery for active nuclear transport. Using sequence homology and prediction algorithms, four putative nuclear localization signals (NLSs) were identified. NOL7 deletion constructs and cytoplasmic pyruvate kinase (PK) fusion proteins confirmed the functionality of three of these NLSs. Site-directed mutagenesis of PK fusions and full-length NOL7 defined the minimal functional regions within each NLS. Further characterization revealed that NLS2 and NLS3 were critical for both the rate and efficiency of nuclear targeting. In addition, four basic clusters within NLS2 and NLS3 were independently capable of nucleolar targeting. The nucleolar occupancy of NOL7 revealed a complex balance of rapid nucleoplasmic shuttling but low nucleolar mobility, suggesting NOL7 may play functional roles in both compartments. In support, targeting to the nucleolar compartment was dependent on the presence of RNA, as depletion of total RNA or rRNA resulted in a nucleoplasmic shift of NOL7.

**Conclusions:**

These results identify the minimal sequences required for the active targeting of NOL7 to the nucleus and nucleolus. Further, this work characterizes the relative contribution of each sequence to NOL7 nuclear and nucleolar dynamics, the subnuclear constituents that participate in this targeting, and suggests a functional role for NOL7 in both compartments. Taken together, these results identify the requisite protein domains for NOL7 localization, the kinetics that drive this targeting, and suggest NOL7 may function in both the nucleus and nucleolus.

## Background

NOL7 is a predicted 29 kDa, 257 amino acid protein with no significant homologies to other characterized proteins that localizes to the nucleus and nucleoli of cells. NOL7 localizes to 6p23, a region with frequent loss of heterozygosity (LOH) in a number of cancers, including hormone-refractory breast carcinoma, leukemia, lymphoma, osteosarcoma, retinoblastoma, nasopharyngeal carcinoma and cervical cancer (CC) [1-19]. Using CC as a model for investigation, where LOH of 6p23 is the most common allelic loss in this neoplasm [20-25], we demonstrated that reintroduction of NOL7 suppresses *in vivo *tumor growth by 95% [26]. This suppression is due in part to the induction of an anti-angiogenic phenotype via decreased expression of the angiogenic factor Vascular Endothelial Growth Factor (VEGF) and increased expression of the inhibitor of angiogenesis Thrombospondin-1 (TSP-1).

One of the important features that differentiate eukaryotic from prokaryotic cells is the presence of intracellularly distinct compartments and organelles such as the nucleus, nucleolus and mitochondria. The rapid exchange of proteins between the cytoplasm and the nucleus is a vital process in eukaryotic cells, and this occurs through the nuclear pore complex (NPC), a large macromolecular structure embedded in the double membrane of the nuclear envelope [27-29]. Small molecules such as ions and some small proteins can move from the cytoplasm to the nucleus by passive diffusion through the NPC. However, proteins larger than ~20 kDa typically cross the NPC in a carrier-mediated fashion [30]. This active nuclear transport of proteins is mediated by specific amino acids sequences, which are referred to as nuclear localization signals (NLS) and nuclear export signals (NESs). The classical NLSs contain a cluster of basic amino acids, while classical NESs contain stretches of hydrophobic, leucine-rich residues. The best-described nuclear import pathway is driven by the so-called classical NLS (cNLS). This signal is typically lysines (K) or arginines (R), that are organized as a single-stretch monopartite NLS: (K/R)_4-6_, or as a bipartite NLS in which there are two small clusters typically separated by ten to twelve amino acids (K/R)_2_X_10-12_(KR)_3_. The SV40 large-T antigen (PKKKRKV) and nucleoplasmin (KRPAATKKAGQAKKKK) cNLS are the prototypical mono- and bipartite cNLS [31,32]. Recently, a tripartite NLS, consisting of three clusters of basic residues separated by two spacer peptides, has also been described [33-38]. Finally, a fourth type of NLS contains two to four dispersed basic residues contiguous to hydrophobic amino acids [39,40]. The active transport of proteins between the cytoplasm and nucleus is facilitated by the karyopherin/importin family of carrier proteins. During classical nuclear import, NLSs are typically recognized in the cytoplasm by a heterodimeric complex consisting of importin α and β with the α-subunit providing the NLS binding site. The NLS protein-receptor complex docks to the nuclear pore complex via importin β and is subsequently translocated through the pore by an energy-dependent mechanism [41]. Once the import complex reaches the nucleus, it is dissociated by RanGTP. Binding of RanGTP to importin β can cause a conformational change, resulting in the release of the importin α/cargo complex [42]. Recent modeling studies have shown that some cargo proteins can also bind directly to β -karyopherins [43].

While active transport mechanisms are required for nuclear localization, targeting to the nucleolus has been shown to depend on interactions with nucleolar constituents. Nucleolar localization sequences (NoLSs) have been shown to represent binding domains with resident nucleolar proteins, rRNA, and other nucleolar components and function more as retention rather than targeting signals [44-50]. The affinity and stability of these interactions among nucleolar proteins has been shown to have functional consequences that are reflected in the dynamics of their nucleolar localization. Many ribosomal proteins show higher immobility and slower recovery compared to processing and transcriptional factors, and these differences have been attributed to the stability and duration of their nucleolar functions [51,52]. Mutations, truncations, and changes in posttranslational modifications that have functional consequences have also been shown to affect the immobility and recovery of classic nucleolar proteins such as NPM [53]. Together, these observations suggest the nucleolar localization is a factor of the affinity, stability, and abundance of nucleolar interactions and that the dynamics of nucleolar occupancy are a reflection of potential functions within the nucleus and nucleolus.

The purpose of this work was to determine the mechanism by which NOL7 was transported into the nucleus, identify the minimal functional sequences required for NOL7 nuclear translocation, and the relative influence each of these sequences may have on the rate and efficiency of localization. We further wished to define the elements responsible for the nucleolar localization of NOL7 and characterize the dynamics of this targeting. Together, the aim of this paper was to define the mechanism responsible for localization of NOL7 within the cell and the functional consequences of the different components that comprise that mechanism.

## Results

### NOL7 is imported into the nucleus via an energy-dependent, nucleoporin-mediated mechanism

**Figure 1:**
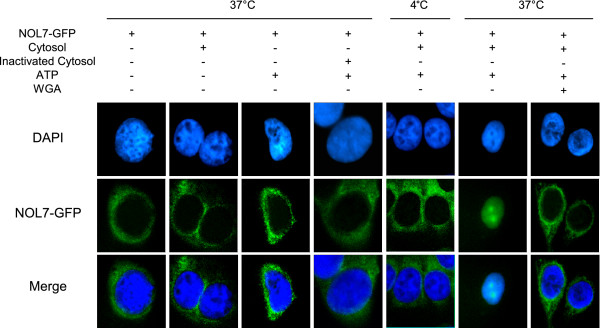
**NOL7 requires cytosolic factors for efficient nuclear localization**. HeLa cells permeabilized with digitonin were incubated at 4°C or 37°C as indicated with (+) or without (-) full length NOL7 expressing a C-terminal GFP tag, cytosol, heat-inactivated cytosol, ATP, or WGA. Localization of NOL7 was confirmed by visualization of GFP.

NOL7 is predicted to have a molecular weight of approximately 29 kDa. Some proteins of this size have been reported to enter the nucleus through passive diffusion, while others require active NLS-mediated transport. To distinguish between an active transport and a passive diffusion mechanism of nuclear localization for NOL7, we subjected GFP-tagged NOL7 to an *in vitro *transport assay using permeabilized HeLa cells [54]. In this assay, the cytoplasmic membranes are permeabilized with digitonin, which depletes the cells of their soluble endogenous cytosolic factors while leaving the nuclear membrane intact. In this fashion, the nuclear import of GFP-tagged proteins can be studied under various conditions. In these experiments, digitonin-permeabilized HeLa cells were incubated with GFP-tagged NOL7 under various conditions to assay the mechanism of its localization. NOL7-GFP alone was not sufficient for nuclear import (Figure 1, first panel). Addition of cytosolic extract or ATP alone was also insufficient for transport (Figure 1, second and third panels), suggesting that NOL7 localization is energy and complex dependent. At 4°C or in the presence of heat-inactivated cytosol, NOL7-GFP was restricted to the cytoplasm (Figure 1, fourth and fifth panels), demonstrating that NOL7 requires cytosolic proteins and is localized via an active transport mechanism. At physiologic temperature, combination of cytosol and ATP was sufficient for NOL7 nuclear localization (Figure 1, sixth panel). However, this was blocked by pretreatment with wheat germ agglutinin (WGA) (Figure 1, seventh panel). Together, these controls demonstrate that NOL7 is actively targeted to the nucleus and nucleolus via an energy-dependent, nucleoporin-mediated mechanism.

### Protein prediction programs identify distinct biochemical domains and putative NLSs in NOL7

**Figure 2:**
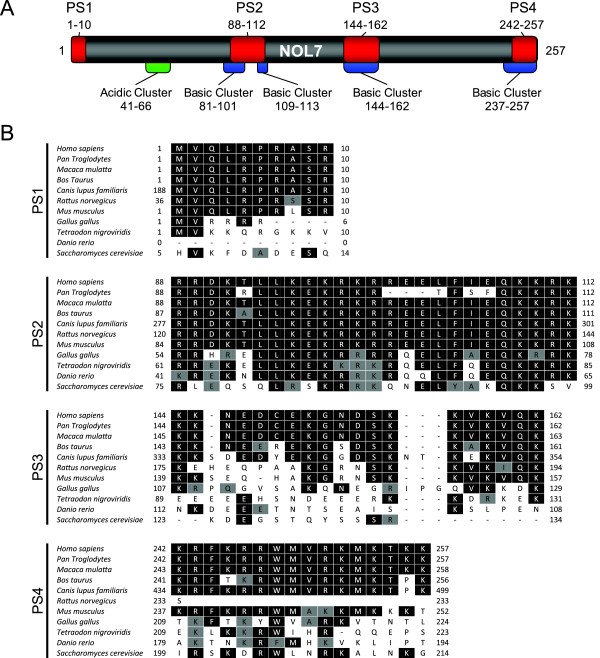
**NOL7 is composed of distinct biochemical domains and multiple putative NLSs that show evolutionary conservation**. (A) Multiple sequential analysis programs confirmed the existence of four basic (blue) and one acidic (green) region in the full-length sequence of NOL7. Putative NLSs identified in sequence analysis programs are shown in red. (B) Sequence conservation between human NOL7 and its putative orthologs was analyzed for each of the putative NLSs and the alignment is shown. Black shaded boxes indicate identical amino acid conservation, while grey boxes signify similar amino acids to *Homo sapiens*. Numbers correspond to residues within the RefSeq sequences listed in the Materials and Methods 2.7.

Database analysis of the full length sequence of human NOL7, utilizing the protein domain prediction programs PSORT [55], TargetP [56], SAPS [57] and NetNES [58], identified five distinct biochemical domains and four putative NLSs within NOL7 (Figure 2). SAPS analysis revealed the existence of four highly basic regions and one acidic domain which correlates with its high pI of 9.7. Stretches of highly basic residues have been shown to participate in nucleic acid binding, nuclear transport, and may contribute to the tumor suppressive function and localization of NOL7 (Figure 2A). While NOL7 lacks homology to other known proteins and domains, the significant sequence conservation among its orthologs suggests a consistent evolutionary role. In particular, four long stretches of basic amino acids are particularly conserved throughout evolution (Figure 2B). Not coincidently, the prediction programs also identified potential NLSs within these basic biochemical domains. However, NOL7 was not predicted to contain any NESs. Putative Sequence (PS) 1, amino acids 1-10 appeared to be a monopartite NLS, while PS2, amino acids 88-112 was predicted to be an example of the recently described tripartite NLS. PS3, amino acids 144-162 appeared to be bipartite NLS and PS4, amino acids 242-257, was predicted to be a bipartite NLS (Figure 2B).

Analysis of potential sequence conservation of each of the four putative NOL7 NLSs among its orthologs was performed using BLAST and aligned by the ClustalW method (Figure 2B). Significant evolutionary conservation between mammalian species *Homo sapiens, Pan troglodytes, Macaca mulatta, Bos taurus, Canis familiaris, Rattus norvegicus*, and *Mus musculus *was observed not only for the NLS regions for the full-length protein as well. Less striking but still significant homology was also seen in *Gallus gallus, Tetraodon nigroviridis, Danio rerio*, and *Saccharomyces cerevisiae*. While some stretches of the full-length protein showed typical divergence of the sequence, the basic stretches of residues that comprised the putative NLSs were remarkably conserved. In particular, PS1, which is composed of three basic residues contiguous to hydrophobic amino acids, showed highly significant homology between all of the NOL7 orthologs. This suggests that PS1 may target NOL7 to the nucleus despite its dispersed amino acid sequence.

Interestingly, no homology could be detected between NOL7 and any other proteins or domains. Further, despite the significant conservation between human NOL7 and its putative orthologs, no functional role can be extrapolated for NOL7. With the exception of S. cerevisiae ortholog Bud21, no functional data exists for any of these proteins. In the case of the putative yeast ortholog, initial studies suggest that the U3 snoRNA function of Bud21 is not conserved, as NOL7 is incapable of interacting with major Bud21 cofactors that regulate its activity (unpublished data). Further, no similarity could be detected between NOL7 and other characterized proteins of any species, either in the context of the full-length NOL7 or for shorter stretches of the protein. Taken together, this data suggests that NOL7 is a critical protein in higher eukaryotes that may function in a specialized manner. Furthermore, the correlation between the regions of strongest sequence conservation and predicted NLSs suggests the localization and function of NOL7 may be linked.

### NOL7 contains three functional NLSs that translocate cytoplasmic PK into the nucleus

**Figure 3:**
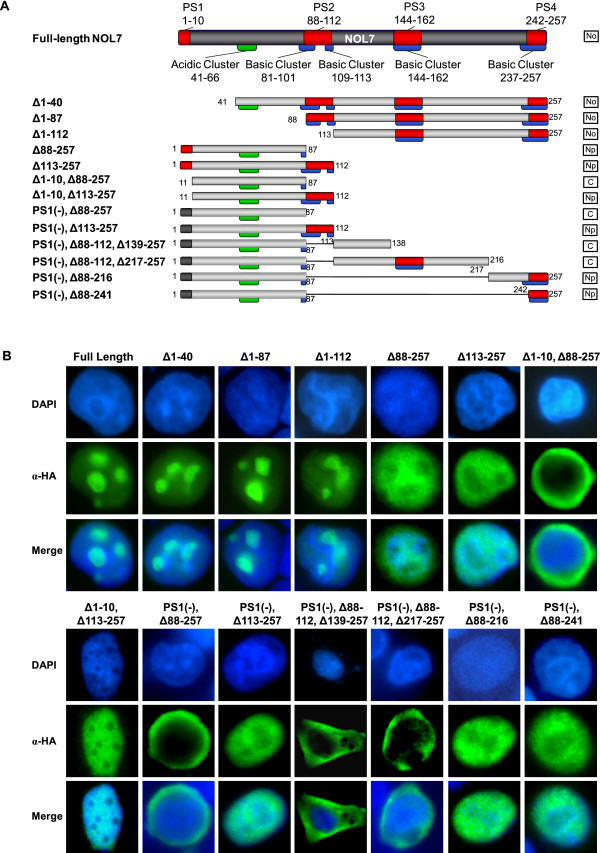
**NOL7 contains three separate NLSs that are necessary for nuclear localization**. (A) Schematic representation of deletion constructs of NOL7 used to determine which regions of NOL7 are required for nuclear localization. Results as demonstrated in (B) are summarized in the column to the right, where "No" designates nucleolar localization, "Np" designates nucleoplasmic localization, and "C" designates cytoplasmic localization. (B) Localization of the constructs was confirmed in HeLa cells by immunofluorescence using an α-HA primary and FITC-coupled secondary antibody.

In order to determine the functionality of the four putative NLSs, we first generated a series of N- and C- terminal deletion mutants of NOL7 with an HA tag that were transiently expressed in HeLa and 293T cells (Figure 3A). The subcellular localization of the truncations was visualized by immunofluorescence with DAPI costaining of the nucleus. While the majority of the deletion constructs retained nuclear localization, a deletion construct lacking all four PSs [Δ1-10, Δ88-257] was cytoplasmic (Figure 3B). Further, PS1 and PS2 were shown to function individually as NLSs, as constructs missing PS2, PS3, and PS4 [Δ88-257] or PS1, PS3, and PS4 [Δ1-10, Δ113-257] were nuclear localized.

While these results demonstrated that PS1 and PS2 were sufficient to target NOL7 to the nucleus, we sought to further clarify the role of PS3 and PS4. Therefore, five additional truncations were cloned together with inactivating mutations in the region of PS1 [PS1(-)]. PS4 was found to be sufficient for nuclear localization of NOL7 [PS1(-),Δ88-216 and PS1(-),Δ88-241] but a construct containing only an intact PS3 [PS1(-),Δ88-112,Δ217-257] remained in the cytoplasm, suggesting that the putative sequence of PS3 is not a functional NLS.

**Figure 4:**
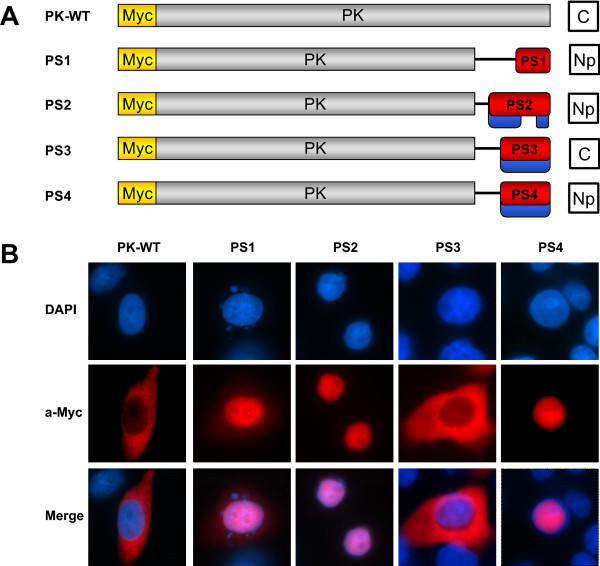
**NOL7 contains three NLSs that are sufficient for nuclear localization**. (A) Schematic representing the three different NLSs cloned in-frame with the cytoplasmic protein PK bearing a c-myc tag. Results demonstrated in (B) are summarized in the column to the right, where "Np" designates nucleoplasmic localization and "C" designates cytoplasmic localization. (B) Localization of the constructs in Hela cells was confirmed by immunofluorescence using an α-myc primary and Cy-3 conjugated secondary antibody. Costaining of the nucleus with DAPI is shown in blue.

To specifically determine if the three candidate NLSs were sufficient to target proteins for nuclear import and further confirm that PS3 was not a functional NLS, the localization of a series of fusion constructs containing each putative NLS sequence and the cytoplasmic protein PK was evaluated. PS1, 2, 3 and 4 were cloned in frame with the C- terminus of the PK bearing an N-terminal myc tag and transiently transfected into HeLa cells (Figure 4A). The subcellular localization of the chimeric proteins was visualized using an α-myc monoclonal antibody and Cy3-conjugated secondary, with DAPI costaining for visualization of the nucleus. Both wild-type Myc-tagged PK protein and the PS3-PK fusion were seen exclusively in the cytoplasm (Figure 4B). In contrast, PK-PS1, PK-PS2, and PK-PS4 localized predominately to the nucleus (Figure 4B). Taken together, these results from the truncation and PS-PK fusion experiments confirm that PS3 is not a functional NLS while PS1, PS2, and PS4 are functional nuclear localization signals. Furthermore, the data demonstrate that each NLS is capable of translocating a cytoplasmic protein into the nucleus independently. From this point forward, we will therefore refer to PS1, PS2, and PS4 as NLS1, NLS2, and NLS3, respectively.

### Identification of the amino acids required for functionality of each NLS

**Figure 5:**
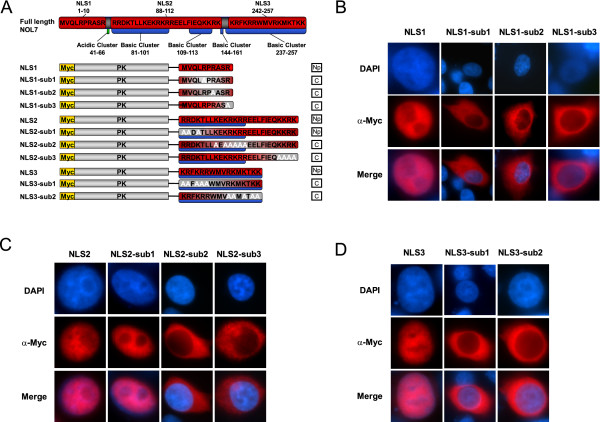
**Basic residues within each of the NLSs are required for nuclear localization of PK**. (A) Schematic representing the three different NLSs bearing neutralizing mutations in the basic residues were cloned in-frame with the cytoplasmic protein PK bearing a c-Myc tag. Results demonstrated in (B-D) are summarized in the column to the right, where "Np" designates nucleoplasmic localization and "C" designates cytoplasmic localization. (BD) Subcellular localization was determined by immunofluorescence in HeLa cells using an α-myc primary and either a HRP or Cy-3 conjugated secondary antibody. Costaining of the nucleus with DAPI is shown in blue. (B) Mutations in either the first (NLS1-sub1), second (NLS1-sub2), or third (NLS1-sub3) basic cluster of NLS1 were mutated in alanine and visualized for nuclear localization. (C) Expression and localization of NLS2 mutants lacking either the first (NLS2-sub1), second (NLS2-sub2), or third (NLS2-sub3) basic cluster. (D) Expression and localization of the constructs bearing mutations in the first (NLS3-sub1) or second (NLS3-sub2) basic cluster of NLS3.

The deletion and PK fusion constructs demonstrated the regions within NOL7 that are individually capable of driving nuclear localization, but do not define the specifc sequence elements that comprise the individual NLSs. To specifically define the minimal amino acids required for functionality of each NLS, site-directed mutagenesis was performed to convert the basic amino acids of interest (arginine and lysine) to the nonpolar, electrically neutral amino acid alanine in each of the NLSs. Within each sequence, three individual basic amino acids (NLS1) or clusters of basic amino acids (NLS2 and NLS3) were identified and mutated individually to determine their relative contribution to the functionality of the NLS. Each intact NLS and the mutant NLSs were cloned in-frame with PK bearing an N-terminal myc tag (Figure 5A). As before, fusion with all three wild type NLSs resulted in nuclear localization of PK (Figure 5B-D). Mutation of any one of the three basic residues of NLS1 abolished nuclear localization, suggesting that each of these amino acids is critical for functionality of NLS1 (Figure 5B). For NLS2, loss of the second or third cluster of basic residues resulted in cytoplasmic localization, while loss of the first basic cluster had no effect on nuclear localization of PK (Figure 5C). These mutations suggest that the minimal region required for nuclear localization directed by NLS2 resides within residues 95-112. Consistent with our predictions, both basic clusters of NLS3 were required for nuclear localization (Figure 5D), suggesting that NLS1 is a monopartite sequence while both NLS2 and NLS3 are bipartite sequences. Taken together, these data confirm the functionality of the NLSs and define the specific amino acids present in each of the individual NLS that are required for the nuclear import of PK.

**Figure 6:**
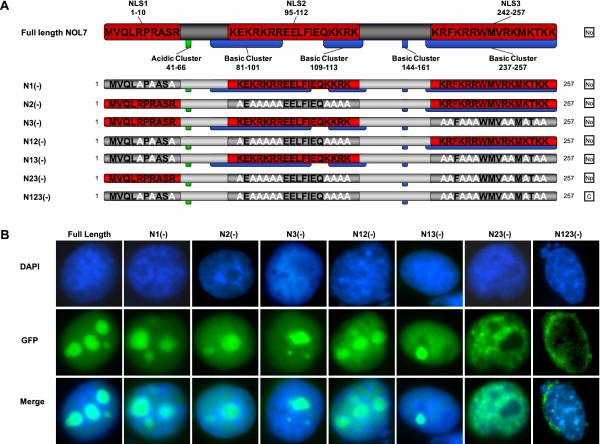
**Basic residues within each of the NLSs are required for nuclear localization of full-length NOL7**. (A) Schematic representing the different mutant constructs used to evaluate nuclear localization in the context of the full length protein. Results demonstrated in (B) are summarized in the column on the right, where "No" designates nucleolar localization, "Np" designates nucleoplasmic localization, and "C" designates cytoplasmic localization. (B) Localization of the GFP-tagged constructs in HeLa cells was confirmed by fluorescent microscopy and costaining of the nucleus with DAPI is shown in blue.

While experiments using individual NLS fused to PK are useful, there are several limitations to these types of studies. For example, Burgess et al [59] demonstrated that EBNA3B has three functional NLSs when investigated in truncation experiments but only two were found to be functional in the context of the full-length protein. To determine the contributions of each NLS within full-length GFP-tagged NOL7, the arginine and lysine residues in each NLS were mutated to alanine (Figure 6A). The subcellular localization of the constructs was visualized using by GFP fluorescence, with DAPI costaining of the nucleus. Mutation of all three NLSs resulted in cytoplasmic localization of NOL7 but retention of only one NLS was sufficient for nuclear localization (Figure 6B). Taken together, these results demonstrate that NOL7 has three functional NLS that can independently cause translocation of full length NOL7.

### Each NLS contributes differentially to the rate and efficiency of NOL7 nuclear import

While each NLS was shown to be independently capable of directing nuclear transport of NOL7, it was unclear what the relative contribution of each signal to this function might be. To address this question, we determined the rate and efficiency of nuclear import for each NLS construct. While fluorescence recovery after photobleaching (FRAP) has been employed previously for measuring the rate of import, this method is limited to small bleaching areas and measures a combination of active nuclear import and nucleoplasmic diffusion, the magnitude of which can vary greatly between proteins [60-62]. In most cases, the nuclear diffusion can be considered equivalent among different constructs of the same protein. However, subnuclear targeting of proteins within the nucleus affects their nuclear diffusion and can no longer be discounted in the calculation of rate by FRAP. As mutant constructs of NOL7 are differentially localized to the nucleoplasm and nucleolus, a different approach needed to be applied to investigate the role of different NLSs in the rate of nuclear import that would not be influenced by mobility within the nuclear compartment. Therefore, two complementary methods were adapted to measure both rate and efficiency of NOL7 nuclear import, based on quantitative immunofluorescence methods previously established in the literature [63-67]. In both cases, HA-tagged NOL7 constructs were transfected into HeLa cells and imaged by immunofluorescence against the HA tag. Using ImageJ software, the fluorescence intensity was measured and reported as a ratio of nuclear to total fluorescence. While previous reports typically utilize the nuclear to cytoplasmic ratio, we normalized to total cell fluorescence to accommodate differences in subnuclear localization and expression level between the different mutant constructs.

**Figure 7:**
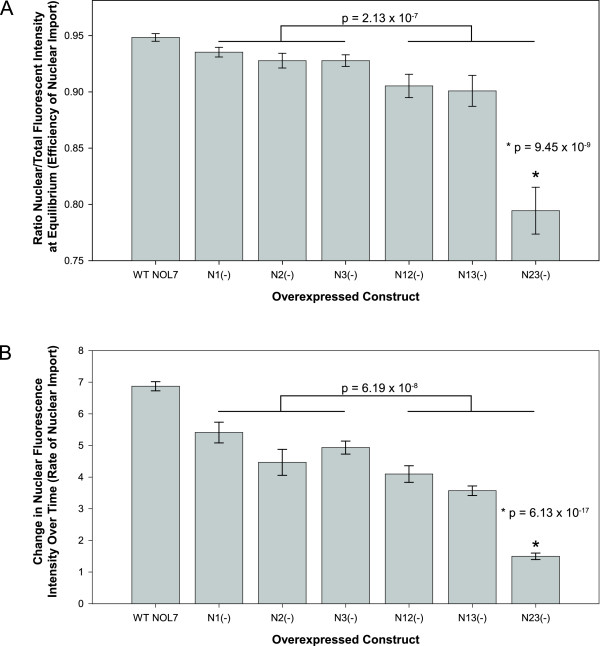
**Each NLS contributes differently to the rate and efficiency of NOL7 nuclear localization**. The steady-state efficiency and rate of import for NLS mutants was evaluated to determine their relative contribution to the subcellular localization of NOL7 in HeLa cells. (A) Twenty hours after transfection, mutants were imaged by immunofluorescence against the HA tags and costained with DAPI and WGA to delineate the nucleus and cytoplasm. Using ImageJ, the nuclear-to-total cell fluorescence ratio was calculated for twenty cells per construct. Error bars represent standard error. (B) Cells were transfected with the different NOL7 NLS constructs and imaged at 5, 6, 7, and 8 hours post-transfection. The nuclear accumulation was measured by α-HA immunofluorescence and the rates were calculated as the change in nuclear signal over time. Bars represent the average rate for ten cells and error bars are representative of the standard error.

For the efficiency experiments, data was collected twenty hours after transfection, when import had reached steady-state equilibrium (Figure 7A). It was found that WT NOL7 was most efficiently localized to the nucleus, and the strictly nucleoplasmic mutant N23(-), was least efficiently targeted. The single mutants demonstrated nearly 10% more efficient nuclear targeting than the double mutants, with a p-value of 2.13×10^-7^. The most dramatic loss in targeting efficiency was observed upon the combined loss of NLS2 and NLS3, with over a 15% decrease in efficiency for N23(-) compared to WT NOL7 and over 10% decrease compared to all other mutants. This decrease was highly significant, with a p-value of 9.45×10^-9 ^compared to WT NOL7 and 2.68×10^-5 ^and 1.24×10^-4 ^compared to the other double mutants N12(-) and N13(-), respectively. Together, these observations suggest that NLS2 and NLS3 are the major sequences involved in the efficient targeting of NOL7 to the nucleus.

To determine the rate of nuclear import, a similar approach was used, this time measuring the relative nuclear fluorescence intensity over a four-hour time course. To ensure that this rate represented strictly nuclear import, the increase in fluorescence intensity was measured prior to the establishment of steady-state NOL7 levels. To determine the time frame for measurement, we transfected cells with GFP-tagged wild type NOL7 and measured the time posttransfection when fluorescent signal can first be detected until it accumulates to a steady-state level. It was determined that NOL7 protein is detectable 5 hours posttransfection, and its accumulation reaches steady-state levels approximately 10 hours after transfection (Additional file 1: Supplementary Movie 1). Therefore, the rate was calculated as the change in fluorescence intensity at 5, 6, 7, and 8 hours posttransfection (Figure 7B). Loss of even one NLS had a significant effect on the change in rate, regardless of identity. The single NLS mutants had a significantly higher rate of import than the double mutants (4.94 ± 0.34 versus 3.05 ± 0.40, p = 6.19×10^-8^). The most dramatic decrease was observed with the N23(-) mutant, which was imported at a rate nearly 80% less than WT NOL7, with a p-value of 6.13×10^-17^. This suggests that each NLS plays a unique role in the targeting of NOL7. Together with efficiency, this suggests that NLS2 and NLS3 in combination are critical for efficient and rapid targeting of NOL7 to the nucleus.

### NLS2 and NLS3 of NOL7 comprise domains that are required for nucleolar localization

**Figure 8:**
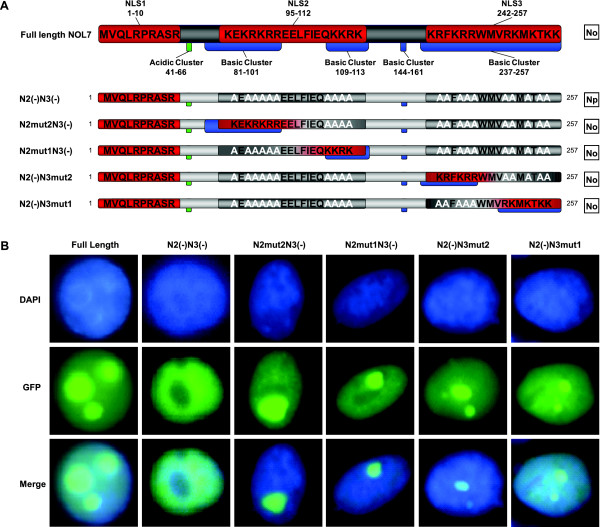
**Basic residues within NLS2 and NLS3 are required for nucleolar localization of NOL7**. Basic residues within each of the NLSs are required for nucleolar localization of full length NOL7. A) Schematic representation of the different mutant constructs used to evaluate nucleolar localization. Results demonstrated in (B) are summarized in the column on the right, where "No" designates nucleolar localization and "Np" designates nucleoplasmic localization. B) Localization of the constructs in HeLa cells was confirmed by GFP visualization. Costaining of the nucleus with DAPI is shown in blue.

It has been previously demonstrated that NOL7 localizes to the nucleolus via colocalization with the nucleolar protein NPM [26]. In the analysis of the NLSs, it was noted that loss of NLS2 and NLS3 together abolished the nucleolar but not nuclear localization of NOL7 (Figure 6B). Interestingly, NLS23(-) also significantly decreased both the rate and efficiency of NOL7 nuclear transport (Figure 7A and 7B). To identify the possible NoLS(s) within these signals, systematic mutation of the basic residues within NLS2 or NLS3 was undertaken (Figure 8A). Restoration of any basic cluster in NLS2 or in NLS3 was sufficient to restore nucleolar localization, suggesting that these regions are capable of individually functioning as NoLSs (Figure 8B). Thus, NOL7 contains at least four separate NoLSs within its nuclear targeting sequences that are individually capable of directing nucleolar localization.

### NOL7 demonstrates rapid recovery but low mobility within the nucleolus

**Figure 9:**
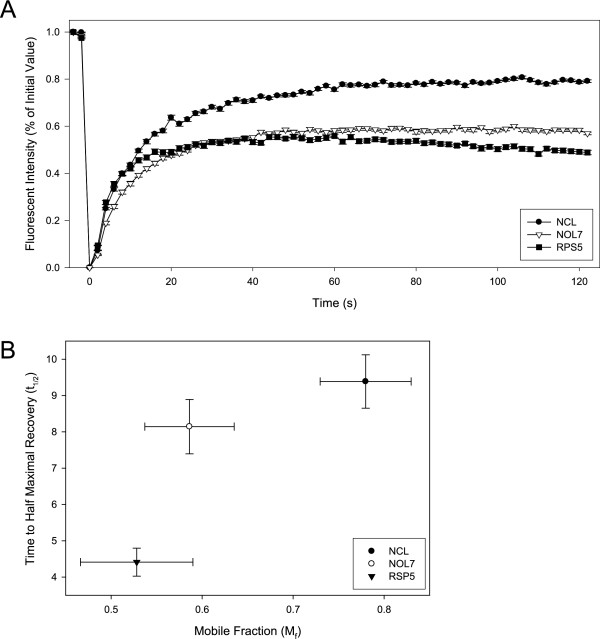
**FRAP analysis of NOL7 nucleolar occupancy demonstrates rapid recovery but low mobility within the nucleolus**. (A) The fluorescence recovery within the nucleolus was measured over time for HeLa cells transfected with GFP-tagged NOL7, the high mobility shuttle NCL, and the low mobility resident protein RPS5. Measurements represent thirteen different cells per protein. The curves were fit to the line curve F(t) = F_∞_(1-e^τ·t^). (B) The nucleolar occupancy was plotted as a function of recovery versus mobility. The mobile fraction was used as a measurement of free versus complexed protein within the nucleolus and calculated from the regression values in (A) using the formula M*_f _*= (F_∞_-F_0_)/(F_i_-F_0_). The half-time to maximal recovery was calculated using the formula t_1/2 _= ln(0.5)/τ and used as a measurement of shuttling between the nucleolus and nucleoplasm. All error bars represent the standard error of the measurement.

Protein occupancy and complex assembly in subnuclear bodies has been shown to relate to function for a majority of proteins [68-70]. Therefore, the nucleolar occupancy of NOL7 was evaluated by FRAP. The occupancy was described by the recovery half life (t_1/2_) and mobile fraction (M_f_) of GFP-fusion constructs. In order to define an upper and lower limit for nucleolar protein mobility, NOL7 was evaluated with the controls NCL, a freely diffusing nuclear/nucleolar shuttle with functions in both the nucleus and nucleolus, and RPS5, a low-mobility resident nucleolar protein (Figure 9A). These proteins represent typical controls within the literature and allow for comparison to other dynamic studies [71]. The t_1/2 _of NOL7 was found to be most similar to a shuttling protein such as NCL, suggesting that NOL7 can freely exchange with the nucleoplasm (Figure 9B). Conversely, the immobile fraction (M_f_) of NOL7 was found to be most similar to an immobile, complexed nucleolar protein like RPS5 (Figure 9B). This is consistent with previous reports describing the nucleolar occupation of a number of nucleolar proteins, including NPM, NCL, and RPS5 [71,72]. This suggests that a large pool of nucleolar NOL7 is functionally occupied in a nucleolar complex, while the free protein is able to shuttle rapidly between subnuclear compartments. Compared to literature reports, this data indicates that NOL7 is most similar to proteins with multiple nuclear and nucleolar roles like NPM, which is both a nucleolar shuttle and associates in functional nucleolar complexes, than either NCL or RPS5. Further, these data suggest that NOL7 shuttles between the nucleolus and nucleoplasm and may play a functional role in both compartments.

### NOL7 localization is dynamically regulated by changes in RNA composition

**Figure 10:**
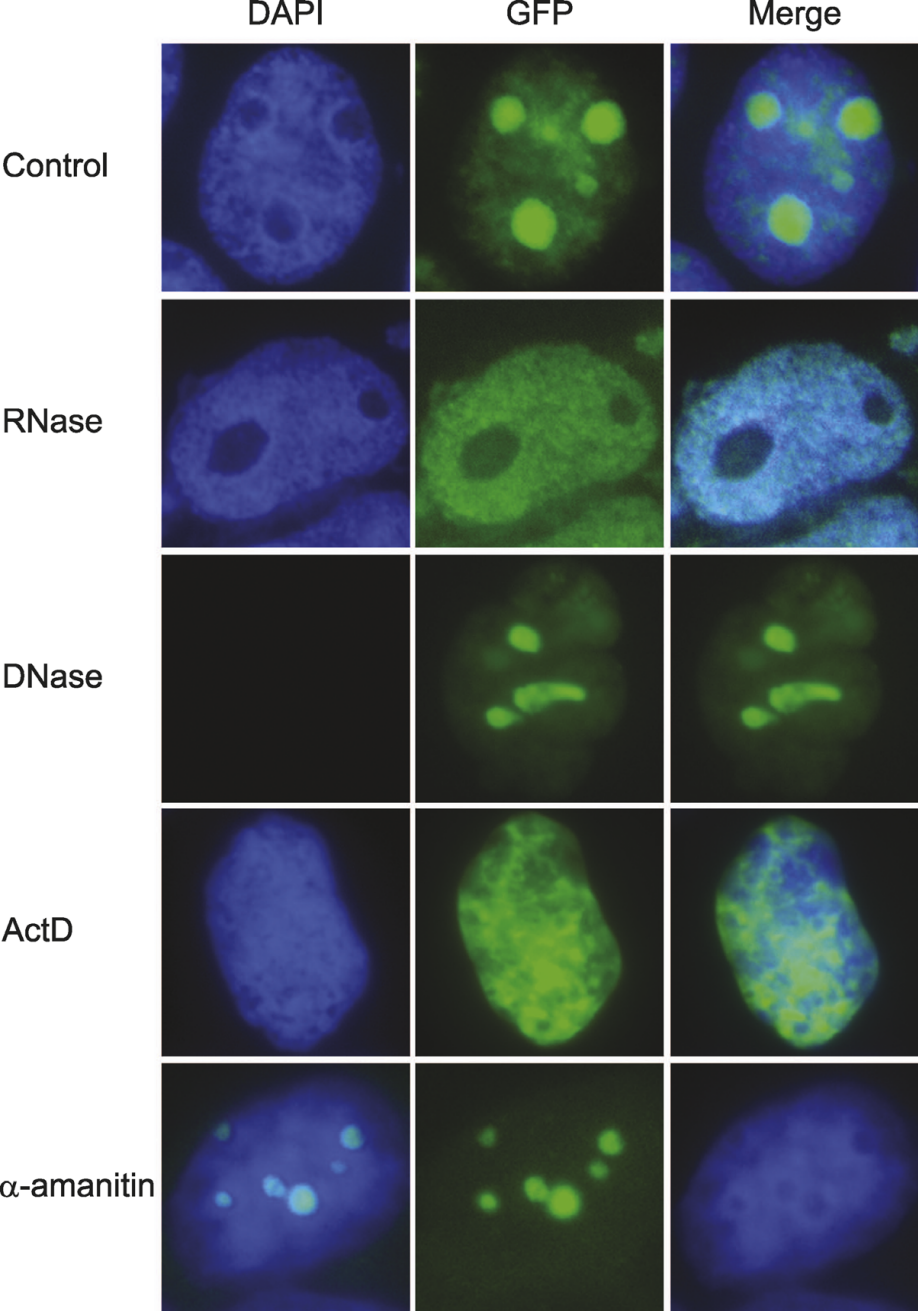
**NOL7 subnuclear localization is dynamically regulated by changes in RNA composition**. 293T cells were stably transfected with NOL7-GFP and treated with RNase A (100 μg/ml, 2 hours), DNase I (100 μg/ml, 2 hours), actinomycin D (0.05 μg/ml, 4 hours), or α-amanitin (50 μg/ml, 4 hours) to specifically deplete individual nucleic acid species. Treatment with DNase (total DNA), RNase (total RNA), ActD (rRNA), or α-amanitin (mRNA) was performed and localization of NOL7 was confirmed by fluorescent microscopy of the GFP tag.

The shuttling of NOL7 between the nucleus and nucleolus suggested that specific interactions within these compartments may regulate the nucleolar occupancy of NOL7. Due to the highly basic nature of the protein, it was hypothesized that subnuclear localization of NOL7 may be due to interactions with nucleic acids. To investigate, various cell treatments were employed to change the abundance of different nucleic acid species (Figure 10). Cells overexpressing GFP-tagged NOL7 were treated with RNase, DNase, actinomycin D (ActD), or α-amanitin and visualized by fluorescence microscopy for changes in subcellular localization. RNase treatment resulted in nucleolar loss and nucleoplasmic accumulation of NOL7, while cells treated with DNase did not show any significant change. Culture of mammalian cells in low doses of ActD selectively inhibit rRNA synthesis while having no effect on tRNA, 5S rRNA, nuclear RNA and mRNA synthesis [73,74]. Similarly, treatment with low doses of α-amanitin inhibits RNAPII and subsequent mRNA synthesis without affecting the abundances of other RNA species. Loss of these specific RNA species has been shown to selectively deplete their RNA-binding protein counterparts from different cellular compartments, enabling visualization of binding activities that may participate in protein localization [75-77]. Upon treatment with ActD, NOL7 was found to translocate to the nucleoplasm. Upon treatment with α-amanitin, no change in the nucleolar localization of NOL7 was observed. However, the nucleoplasmic localization of NOL7 previously observed was absent. This data suggests that targeting of NOL7 to both the nucleus and nucleolus results in multiple RNA-dependent interactions.

## Discussion

Active nuclear transport involves complex interactions between the transport machinery and protein cargo, mediated in part through NLSs. Typically composed of discrete patterns of basic residues, these sequences are recognized by the transport machinery and can vary in their affinity, rate, and efficiency of localization, which in turn can influence the function and biologic relevance of the cargo protein in different physiologic settings. Here, we have shown that NOL7 is targeted to the nucleus via an energy- and nucleoporin-dependent mechanism. This transport is mediated by three evolutionarily conserved but distinct NLSs. In addition, each NLS was found to be independently capable of directing the nuclear localization of the cytoplasmic protein PK or full length NOL7. Each NLS individually and additively contributed to the rate and efficiency of NOL7 nuclear targeting, suggesting that each of the NLSs has differential effects in driving the localization kinetics, likely reflecting differences in the regulation of import. Taken together, these data indicate that NOL7 localization is tightly regulated and may contribute to functions in various cellular compartments.

The transport of proteins and RNAs into the nucleus occurs through the NPC and is an important step in regulating the subcellular location of a number of different proteins, including transcription factors, signalling proteins, and various enzymes. Although alternative mechanisms exist, the classic nuclear import pathway appears to be the predominate method of transport into the nucleus. A recent survey of *Saccharomyces cerevisiae *screened over 5800 genomic sequences and found that 45% contained classic NLSs and nearly 60% of nuclear proteins contained monopartite or bipartite sequences [78]. This is likely true across species, as a number of studies have found that the nuclear transport machinery for essential proteins is highly conserved between animals, yeast and plants [30-32]. This observation is certainly true for the three NLSs present in NOL7, where sequence alignment of the three NLSs demonstrated significant evolutionary conservation and aided in the identification of putative targeting sequences. It further suggested that targeting may play a significant role in the regulation and function of NOL7, as these sequences were highly conserved across species but demonstrated little similarity to other proteins or domains.

NOL7's three functional NLSs are located in the N terminus, middle, and C terminus of the protein. While a single functional NLS is sufficient for most proteins, the presence of multiple functional NLSs is seen frequently among proteins whose function is critically determined by its localization. Proteins such as p53 [79], E2F1 [80], c-Abl [81], p14^ARF^, HPV E6 [82], BRCA2 [83], most ribosomal proteins including RPS7 [84,85], b-myb [86], ATF2/c-jun heterodimer [87], PAK-1 [88] and others have been demonstrated to contain more than one NLS. Interestingly, many of these proteins are also implicated in cancer, and aberrant or mislocalized protein plays a significant role in the development and progression of the disease. As such, nuclear localization, and the rate and efficiency at which it occurs, has been shown to have many downstream functional consequences for proteins [89-92].

Terry, *et al*, have proposed a hierarchical regulation to classical nuclear transport via NLSs, with multiple mechanisms acting at the level of the cargo, receptors, and NPC [93]. The existence of multiple NLSs within a single protein may therefore provide a mechanism to exploit these different targeting controls for proteins whose nuclear localization is critical for function [93]. The first level of regulation involves the NPC, and the permeability, stability, and expression of the proteins that comprise this complex can affect the efficiency and targeting of cargo. The existence of multiple NLSs within NOL7 may therefore be used to achieve nuclear localization despite cellular conditions where NPC is less accessible. The next level of regulation involves the transport receptors. Here, differing accessibility, affinity, competition, and expression of the importins in various cell types and under different cellular conditions can affect transport [40,94-99]. In this case, the existence of multiple NLSs can increase likelihood of transporter interaction regardless of environment, coordinate for better efficiency and rate of localization, or outcompete other NLS-bearing proteins for these receptors. Indeed, combined loss of NLS2 and NLS3 significantly impact both the rate and efficiency of NOL7 localization, and the presence of more than one NLS results in a statistically significant increase in NOL7 nuclear accumulation (Figure 7). Finally, at the level of the cargo, modifications and interactions of the cargo protein itself regulates its own localization. Inter- and intramolecular interactions can provide or preclude access to NLSs, and modifications within NLSs can also affect transport, either inhibiting or promoting import to the nucleus [79,100]. The differential rate and efficiency of localization observed among NOL7 mutants, particularly in the N23(-) mutant, suggests each NLS may participate in different levels of this regulation. In addition, many NLSs have also been shown to harbor subnuclear targeting sequences such as NoLSs. NoLSs typically represent interaction motifs between nucleolar constituents, making nucleolar localization a dynamic, multidirectional process compared to nuclear targeting [44-50,101,102]. Our results have shown that NLS2 and NLS3 include four NoLSs. These sequences are composed of basic clusters and each is capable of individually driving nucleolar localization of NOL7. Whether these regions represent unique binding domains or are functionally redundant to ensure efficient interaction with nucleolar cofactors is unknown at this time.

Investigation of the nucleolar occupancy of proteins under various cellular conditions has demonstrated that the kinetics are often highly similar for functionally related proteins [75]. In particular, FRAP analysis of the recovery and mobility of proteins within this compartment has been shown to reflect their functional roles. The nucleolar mobility is typically viewed as a reflection of the stability of the interactions and size of the interaction complex within that compartment, while the recovery reflects the shuttling characteristics of a protein between the nucleoplasm and nucleolus. While many ribosomal proteins are highly immobile within the nucleolus, proteins such as NCL, UBF, and NPM have higher mobility and rapid recovery, due to their multiple functional roles in the nucleus and nucleolus [60,62,71,72]. FRAP analysis of the nucleolar occupancy of NOL7 demonstrates that a large fraction of nucleolar NOL7 is involved in a relatively stable complex, as evidenced by its small M_f_. Interestingly, free NOL7 protein rapidly shuttles between compartments. These dynamics, with low M_f _and high t_1/2_, have been demonstrated in the literature to be unique to proteins that functionally interact with ribonucleoproteins (RNPs) in both the nucleus and nucleolus such as NPM [60-62,68-72]. Together, this suggests that NOL7 may interact in RNP complexes in both compartments. Further support for the potential nuclear and nucleolar interactions of NOL7 can be observed by the changes in localization for NOL7 upon specific depletion of nucleic acid species. The pattern of NOL7 expression is significantly altered by loss of RNA but not loss of DNA, suggesting that NOL7 is an RNA-associated protein, either directly or through RNP complexes. Further, changes in rRNA and mRNA abundance affected the abundance of NOL7 in the nucleolus and nucleoplasm, respectively, suggesting that NOL7 may be participating in distinct functional complexes within each compartment. Whether this is a direct effect of rRNA and mRNA interaction, or an indirect consequence of changes in the transcriptome of the cell remains to be investigated. However, together these observations indicate that the RNA abundance within the cell can influence the localization of NOL7 protein, and the dynamics of this localization is similar to the kinetics of proteins that play functional roles in nuclear and nucleolar RNP complexes. While it is unknown what, if any, function NOL7 may have in either compartment, it suggests that its localization is actively regulated and this differential targeting may influence its role in cancer development and progression.

Localization and function within multiple cellular compartments has previously been observed for many proteins. In addition, regulation of protein function through localization mechanisms is known to be employed in multiple cancer signaling pathways, including the Wnt, TGFβ, and Hh pathways. Oncogenes and tumor suppressors such as Rb, c-Myc, p53, VHL, and p14^Arf ^have multiple, different functions depending on their localization or sequestration [103-117]. Our evidence suggests that like many of these oncogenes and tumor suppressors, NOL7 may have be regulated through its subcellular localization, and its targeting may be critically linked to its tumor suppressive activity.

## Conclusions

In summary, we have found that NOL7 requires cytosolic proteins for active transport into the nucleus, consistent with a classical import mechanism. We have identified three functional NLSs within NOL7, each of which is independently capable of directing the nuclear localization of the cytoplasmic protein PK or full length NOL7 and contribute to different degrees to the rate and efficiency of NOL7 nuclear import. Further, these sequences harbor at least four NoLSs that are independently capable of mediating nucleolar localization. The nucleolar occupancy of NOL7 is balanced by its rapid recovery and low mobility, similar to other proteins that play multiple functional roles in both the nucleus and nucleolus. Further, the nucleolar localization of NOL7 is dependent upon the presence of rRNA, while the nucleoplasmic localization of NOL7 is mediated by the abundance of mRNA. This work provides the basis for further investigation into the levels, activity, and mechanism of regulation for NOL7 and elucidation of its role in tumor growth suppression.

## Methods

### Deletion Mutant Constructs

Constructs were cloned as described. Template and primer sequences are listed in Additional file 2: Supplementary Table 1.

#### NOL7 Deletion Constructs

Each deletion construct was cloned by PCR and inserted into the pcDNA3.1/Hygro(+) vector (Invitrogen, Carlsbad CA). For the mutants, residues were mutated using the QuickChange site-directed mutagenesis kit (Stratagene, La Jolla CA).

#### NLS-PK Fusion Proteins

Myc-tagged chicken muscle PK expression DNA was obtained from Gideon Dreyfuss (University of Pennsylvania, Howard Hughes Medical Institute) [118]. It was cloned in frame with individual NLSs to create fusion constructs.

#### GFP-fusion Constructs

Fusion constructs were generated using the GFP Fusion TOPO TA Cloning kit (Invitrogen, Carlsbad CA). Briefly, the full-length NOL7 DNA fragments were TOPO cloned into the plasmid vector pcDNA3.1/NT-GFP-TOPO, and the cloning reaction was transformed into chemically competent cells provided in the kit. The plasmids were purified with Qiagen Plasmid Mini kit (Qiagen, Valencia CA), and sequenced for verification of insert orientation. Mutation of individual residues within the NLSs were constructed by using Quikchange XL site-directed mutagenesis kit (Stratagene, La Jolla CA).

#### NOL7-GFP Purification Construct

For nuclear import assays, NOL7-GFP was cloned with tandem C-terminal GFP-V5-His_6 _tags using the Gatweway cloning system from Invitrogen (Carlsbad, CA). All TOPO and LR cloning reactions were performed as described by the manufacturer. First, wild-type NOL7 was PCR amplified and TOPO cloned into pENTR-SD-D-TOPO. The pENTR-NOL7 construct was transferred to the pcDNA-DEST47 vector, resulting in a C-terminal GFP tag. The NOL7-GFP fusion was PCR amplified and TOPO cloned into the pENTR-SD-D-TOPO vector and this time transferred to the pcDNA-DEST40 vector, thereby expressing NOL7 in frame with a tandem C-terminal GFP-V5-His_6 _tag.

### Tissue Culture

HeLa cells were grown in minimum essential medium supplemented with 10% fetal bovine serum (FBS), 100 μg/ml penicillin and streptomycin. 293T cells were grown in DMEM supplemented with 10% FBS, 100 μg/ml penicillin and streptomycin. Transfections for HeLa, and 293T cells were done using Lipofectin following the manufacturer's directions (Invitrogen, Carlsbad CA) in 75 mm^2 ^dishes when the cells were approximately 80% confluent. Five hours after addition of the DNA precipitate, cells were washed and refed with minimum essential medium or Dubecco's Modified Essential media plus 10% FBS. For stable cell lines, cells were selected in 400 μg/ml G418 (Invitrogen, Carlsbad CA) for three weeks. For transient expression experiments, cell extracts were prepared 20-36 hours after transfection.

### Immunofluorescence

For immunofluorescence staining, cells were plated on 4-well chamber slides and were transfected using Lipofectin for HeLa and 293T cells according to the manufacturer's instructions. Expression of all constructs was validated by western blot. Cells were fixed and stained as previously described [119] thirty-six hours post-transfection unless otherwise stated. Immunostaining was performed using the following primary antibodies: Rabbit α-HA (Invitrogen, Carlsbad CA), 1:4000; Mouse α-c-Myc (Ab-1) (Calbiochem, Gibbstown NJ), 1:500. Secondary antibodies were fluorescein isothiocyanate (FITC) AffiniPure F(ab')_2 _Fragment Goat Anti-Rabbit IgG (H+L) (Jackson ImmunoResearch Labs, West Grove PA), 1:500; Cy3 AffiniPure F(ab')_2 _Fragment Goat Anti-Rabbit IgG (H+L) (Jackson ImmunoResearch Labs, West Grove PA), 1:500. Cells were mounted in DAPI-containing media (Vector Labs, Burlingame CA) according to the manufacturer's instructions. WGA staining (Invitrogen, Carlsbad CA) was performed at a concentration of 5.0 μg/ml according to the manufacturer's instructions.

### Protein Purification

293T cells were transfected with the NOL7-GFP purification construct and positive clones were selected and maintained as described. For purification, approximately 1 × 10^8 ^cells were collected by trypsinization and washed twice with ice cold PBS. Cells were pelleted by centrifugation and resuspended in lysis buffer (50 mM sodium phosphate, pH 7.4; 300 mM NaCl; 1% Triton X-100; Roche Complete EDTA-free protease inhibitor tablet). Cell pellets were sonicated 6 × 30 s at 30% power. Lysates were then cleared by centrifugation and the supernatant was collected and filtered through a 0.45 μm filter. Size-exclusion chromatography was performed using a 0.7 cm × 50 cm Econo-column (Bio-Rad, Hercules CA) that was packed with 5-100 kDA polyacrylamide beads (Bio-gel P-100, 45-90 μM, Bio-Rad, Hercules CA) according to manufacturer's instructions. Fractions of approximately 300 μl were collected and tested for the presence of NOL7 by SDS-PAGE followed by silver staining and western blot using mouse α-V5 monoclonal antibody (Invitrogen, Carlsbad CA). Positive fractions were further purified by affinity chromatography against the His_6 _tag of NOL7 using the ProPur IMAC Kit (Nunc, Rochester NY) under native conditions with 30 mM imidazole washes. The column was washed five times and five elution fractions were collected. NOL7-containing fractions were verified by silver stain and western blot against the V5 tag of NOL7. Positive fractions were concentrated and dialyzed against transport buffer (20 mM Hepes-KOH, pH 7.3, 110 mM potassium acetate, 5 mM sodium acetate, 1 mM EGTA, Roche complete mini protease inhibitor) for use in the import assay.

### Preparation of Cytosol Fractions

Exponentially growing cultures of HeLa cells were collected by low speed centrifugation and washed twice with cold PBS, pH 7.4, by resuspension and centrifugation. The cells were then washed with 10 mM Hepes, pH 7.3, 110 mM potassium acetate, 2 mM magnesium acetate, 2 mM DTT and pelleted. The cell pellet was gently resuspended in 1.5 volumes of lysis buffer (5 mM Hepes, pH 7.3, 10 mM potassium acetate, 2 mM magnesium acetate, 2 mM DTT, 20 μM cytochalasin B, 1 mM PMSF, and 1 μg/ml each aprotinin, leupeptin, and pepstatin) and swelled for 10 min on ice. The cells were lysed with a homogenizer. The resulting homogenates were centrifuged at 1,500×g for 15 min to remove nuclei and cell debris. The supernatants were then sequentially centrifuged at 15,000×g for 20 min and 100,000×g for 30 min. The final supernatants were dialyzed against transport buffer (20 mM HEPES, pH 7.3, 110 mM potassium acetate, 5 mM sodium acetate, 2 mM magnesium acetate, 1 mM EGTA, 2 mM DTT, and 1 μg/ml each aprotinin, leupeptin, and pepstatin) and frozen in aliquots in liquid nitrogen before storage at -80°C.

### Cell Permeabilization and In Vitro Transport Assay

Import assays was performed essentially as previously described [54]. Cells plated on 4-well chamber slides were rinsed in cold transport buffer (20 mM Hepes, pH 7.3, 110 mM potassium acetate, 5 mM sodium acetate, 2 mM DTT, 1.0 mM EGTA, and 1 μg/ml each aprotinin, leupeptin, and pepstain). Wells were immersed in ice cold transport buffer containing 40 μg/ml digitonin (Calbiochem, Gibbstown NJ). The cells were allowed to permeabilize for 5 min, after which the digitonin-containing buffer was removed and replaced with cold transport buffer. For each assay, 150 μl of transport buffer was supplemented with 10 μg/ml NOL7-GFP and incubated for 30 minutes at either 37°C or 4°C. Where indicated, assays were supplemented with 1 mM ATP and 15 mg/ml cytosol. For heat-inactivated cytosol, extracts were boiled at 95°C for 5 min, chilled, and then added to the import assay. For WGA treatment, cells were pre-incubated with 50 ug/ml WGA for 15 minutes at 20°C, washed, and then the import assay was performed as described above. After the 30 min incubation, all slides were washed, fixed with 4.0% paraformaldehyde and analyzed directly by fluorescence microscopy.

### Protein analysis, Domain prediction, and sequence alignment

Protein analysis of NOL7 was carried out using a variety of prediction programs on the following accession sequences: Homo sapiens, NP_057251.2; Pan troglodytes, XP_518245.2; Macaca mulatta, XP_001092572.1; Bos taurus, NP_001029556.1; Canis familiaris, XP_535892.2; Rattus norvegicus, XP_573999.2; Mus musculus, NP_076043.2; Gallus gallus, XP_418926.1; Tetraodon nigroviridis, CAF97792.1; Danio rerio, XP_687281.1; Saccharomyces cerevisiae, NP_014721.1. Alignment of sequences was doing using MegAlign software under the ClustalW parameters.

### FRAP Photobleaching, Imaging, and Quantitation

Approximately forty-eight hours after transfection, HeLa cells were maintained in MEM supplemented with 30 mM Hepes, pH 7.1, to stabilize the pH of the medium during imaging. FRAP was performed on a DM4000 microscope (Leica Microsystems, Wetzlar Germany) equipped with a MicroPoint Laser System (Photonic Instruments, St. Charles, IL), a Roper Coolsnap HQ camera (Princeton Instruments, Trenton NJ), and a Leica 63X HCX PL APO L U-V-I aqueous immersion objective (Molecular Devices, Sunnyvale CA). Fluorescence intensity was measured using Metamorph imaging software (Universal Imaging Corp, West Chester PA). The average intensities of the areas of interest, including before, immediately after, and a series of time points after bleaching, were measured under the same condition for each data set. Data was analyzed using SigmaPlot software and fit to the curve F(t) = F_∞_(1-e^τ·t^). From the regression values, the half-maximal recovery [t_1/2 _= ln (0.5)/τ] and mobile fraction [M_f _= (F_∞_-F_0_)/(F_i_-F_0_)] were calculated for each replicate and statistical significance was determined using Student's t-test.

### Transport Efficiency Experiments

HeLa cells were fixed and stained with rabbit α-HA primary (Invitrogen, Carlsbad CA, 1:4000 dilution) and FITC AffiniPure F(ab')_2 _Fragment Goat Anti-Rabbit IgG (H+L) secondary (Jackson ImmunoResearch Labs, West Grove PA, 1:500 dilution) as described twenty hours after transfection. Immunofluorescent images were captured by using Zeiss Axiovert 200 M microscope system. Image analysis was performed using Image J to quantify per unit area staining intensity in the total cell and nucleus. Twenty high power fields were selected for analysis of each stain. The efficiency was calculated as the ratio of nuclear to total intensity and Statistics were evaluated using Student's t-test.

### Transport Rate Experiments

HeLa cells were fixed and stained with rabbit α-HA primary (Invitrogen, Carlsbad CA, 1:4000 dilution) and FITC AffiniPure F(ab')_2 _Fragment Goat Anti-Rabbit IgG (H+L) secondary (Jackson ImmunoResearch Labs, West Grove PA, 1:500 dilution) as described 5, 6, 7, and 8 hours after transfection. Immunofluorescent images were captured by using Zeiss Axiovert 200 M microscope system. Image analysis was performed using Image J to quantify the nuclear staining intensity per unit of area. Ten high power fields were selected for analysis of each construct. The rate of import was calculated as the slope of the fluorescent intensity versus time and the statistical significance of this data was evaluated using Student's t-test.

### Drug treatment and fluorescence microscopy

HeLa cells were stably transfected with wild-type NOL7-GFP and plated on 2-well chamber slides. When cells were approximately 70% confluent, media was replaced with serum-free DMEM containing 0.05 μg/ml actinomycin D (Sigma-Aldrich, St. Louis MO) or 50 μg/ml α-amanitin (Sigma-Aldrich, St. Louis MO) and incubated for 4 hours at 37°C in 5% CO_2_. Cells were then fixed with 4% paraformaldehyde, washed with PBS, and mounted with DAPI-containing media (Vector Labs, Burlingame CA). For nuclease treatment, cells were first washed with PBS and permeabilized with ice-cold methanol for 10 min and then incubated with 100 μg/ml RNase A (Sigma-Aldrich, St. Louis MO) or 100 μg/ml DNase I (Sigma-Aldrich, St. Louis MO) at 37°C for 2 hours. Cells were then fixed and mounted in the same manner. All cells were imaged on a Zeiss Axioplan microscope.

## Abbreviations

NLS(s): nuclear localization signal(s); CC: cervical cancer; HA: hemagglutinin; PK: pyruvate kinase; CHX: cycloheximide; VEGF: vascular endothelial growth factor; TSP-1: thrombospondin-1; NPM: nucleophosmin; NCL: nucleolin; RPS5: ribosomal protein 5; ribonucleoprotein: RNP; NPC: nuclear pore comples; NES: nuclear export signal; cNLS: classical nuclear localization signal; NoLS: nucleolar localization signal; WGA: wheat germ agglutinin; FBS: fetal bovine serum; FITC: fluorescein isothiocyanate; FRAP: (fluorescence recovery after photobleaching).

## Authors' contributions

GZ performed plasmid construction, localization immunofluorescence, import assays, transport rate and efficiency experiments, and FRAP. CLD performed plasmid construction, sequence analysis, protein purification, drug treatment fluorescent microscopy, statistical analysis, and participated in the design of the study and manuscript preparation. MWL conceived of the study, and participated in its design and coordination and helped to draft the manuscript. All authors read and approved the final manuscript.

## Supplementary Material

Additional file 1 **Supplementary Movie 1 - Detection of NOL7-GFP Posttransfection**. HeLa cells were transfected with WT NOL7 in frame with a GFP fusion tag and imaged every fifteen minutes from the time fluorescent signal can first be detected, approximately five hours after transfection, until it reaches steady-state intensity, approximately nine hours after transfection. Time zero corresponds to five hours posttransfection. Click here for file

Additional file 2 **Supplementary Table 1 - Primers used to clone the constructs used in this study**. Each construct is listed, along with the forward and reverse PCR primers and template for cloning PCR reaction able legend text Click here for file
