# Supplementary figures and images for: Identification and functional analysis of NOL7 nuclear and nucleolar localization signals
Source: BMC Cell Biol. 2010 Sep 27;11:74. doi: 10.1186/1471-2121-11-74 (PMC2957388; doi:10.1186/1471-2121-11-74)

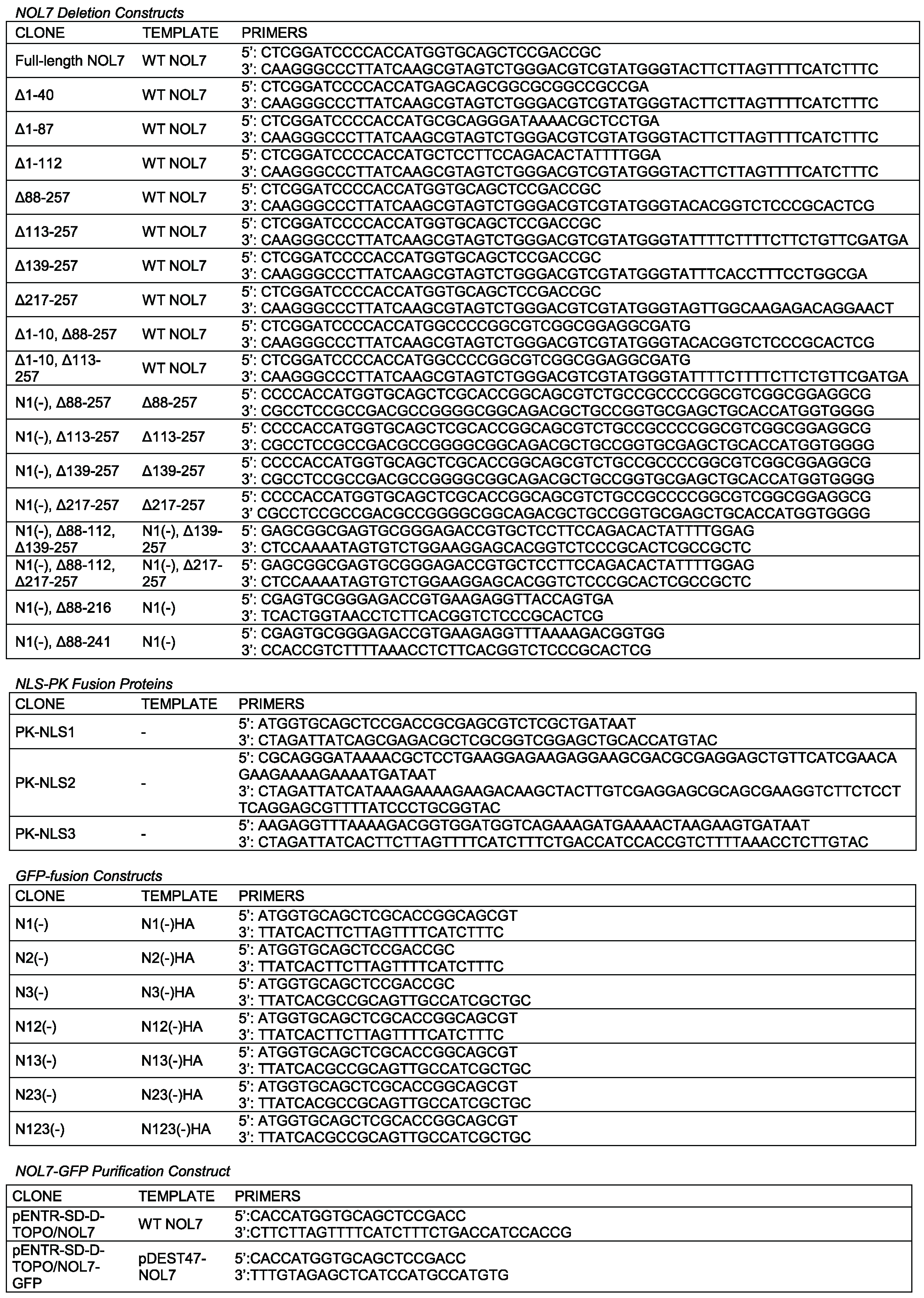

Supplement: Additional file 2 — Supplementary Table 1 - Primers used to clone the constructs used in this study. Each construct is listed, along with the forward and reverse PCR primers and template for cloning PCR reaction able legend text [file 1471-2121-11-74-S2.TIFF]
